# Preparation of β-CD-Ellagic Acid Microspheres and Their Effects on HepG2 Cell Proliferation

**DOI:** 10.3390/molecules22122175

**Published:** 2017-12-08

**Authors:** Hongkai Wang, Yingxia Zhang, Zhongjing Tian, Jing Ma, Meiling Kang, Chengshi Ding, Dongfeng Ming

**Affiliations:** 1 College of Life Science, Zaozhuang University, Zaozhuang 277160, China; whk62@126.com (H.W.); yingxiazh@126.com (Y.Z.); jingzt03@163.com (Z.T.); kmling006@126.com (M.K.); 2 College of Medical Science, Zaozhuang Vocational College, Zaozhuang 277800, China; ma_haiming@163.com

**Keywords:** β-CD-ellagic acid microspheres, preparation, identification, HepG2 cell proliferations

## Abstract

Objective: In this study, β-cyclodextrin (β-CD) was chosen as the coating for ellagic acid to prepare ellagic acid microspheres, and the effect of microspheres on the growth of HepG2 cells was observed. Methods: Scanning electron microscopy, infrared spectroscopy, and release rate analysis were used to identify the formation of ellagic acid microspheres. Methyl thiazolyl tetrazolium (MTT) assay was used to detect the effect of different concentrations of ellagic acid microspheres on tumor cell proliferation at 6, 12, 24 and 36 h, and cell morphology and quantity were observed using hematoxylin-eosin (HE) staining. Single-cell gel electrophoresis was used to observe the effect of ellagic acid microspheres on the DNA damage of HepG2 cells, and the Olive tail moment and the mRNA expression of tumor suppressor protein gene p53 was measured. Results: β-CD could be used as wrapping material of ellagic acid to prepare ellagic acid microspheres. HepG2 cell proliferation could be inhibited by 0.1, 0.3 and 0.5 g/L of ellagic acid microspheres in a dose- and time-dependent manner, and the mechanism of proliferation inhibition was related to DNA damage and cell apoptosis. Conclusion: Preparing ellagic acid microspheres with β-CD is feasible, and ellagic acid microspheres have potential therapeutic value (anticancer).

## 1. Introduction

Pomegranate (*Punica granatum* L.) belongs to the Punicaceae family and originates in Central Asia [1]. Currently, pomegranates are widely grown in China, and the famous production areas include Zaozhuang in Shandong province, Mengzi in Yunnan province, Lintong in Shaanxi province, Kaifeng in Henan province, Huaiyuan in Anhui province, Yecheng in Xinjiang province, and Huili in Sichuan province [2,3]. Its active ingredients are mainly polyphenols, flavonoids, alkaloids, steroids, and triterpene compounds, among which polyphenols have the highest content [4,5]. Ellagic acid is a polyphenol and a dimeric derivative of gallic acid. It shows the tannin structure of trans gallic acid and has antioxidant, antibacterial, and antitumor properties, as well as other biological activities [6,7,8]. In recent years, researchers widely studied the inhibition of ellagic acid in cancer induced by chemicals. In vivo and in vitro, ellagic acid can inhibit tumor cell proliferation and induce cell death, especially for breast cancer, lung cancer, and prostate cancer [9,10,11]. However, ellagic acid is poorly soluble, unstable, easily oxidized by heat, and difficult to absorb; its role is only short term, unlike other polyphenols [12,13,14]. If slow-release ellagic acid microspheres could be prepared, the abovementioned problem could be solved.

Cyclodextrin (CD) is a series of cyclic oligosaccharides produced by cyclodextrin glucose transferase. One of the biggest features of CD is the formation of inclusion compounds [15,16]. According to the number of glucose monomers, CDs are classified into a variety of categories. α-Cyclodextrin, β-cyclodextrin (β-CD), and γ-cyclodextrin are three of the most studied CDs, and they contain six, seven, and eight glucose monomers, respectively. In the molecular structure of CD, glucose monomers linked by the 1,4-glycosidic bond cannot rotate freely, and the hydroxyl stretches out. Thus, CD possesses a cage-like supramolecular structure with a hydrophobic cavity and hydrophilic external; it is able to form inclusion complexes with a wide variety of hydrophobic guest molecules and consequently improves their solubility. The inclusion compound—CDs outside and the guest molecules inside—is similar to a molecular capsule and may have a slow-release effect [17,18,19].

In this study, β-CD was used to parcel ellagic acid extracted from pomegranate rind, and ellagic acid microspheres were prepared. Scanning electron microscopy (SEM), Fourier-transform infrared spectrum (FTIR), and release rate detection were used to identify the formation of ellagic acid microspheres. In vitro, cultured human hepatocellular carcinoma HepG2 cells were treated with different concentrations of ellagic acid microspheres, and MTT (methyl thiazolyl tetrazolium) assay was used to detect the growth of cells at different times. Hematoxylin-eosin staining (HE staining) was used to observe changes in the morphology and number of tumor cells. Single-cell electrophoresis was used to observe the DNA damage of tumor cells. Reverse transcription-quantitative polymerase chain reaction was used to measure the mRNA expression of p53 in tumor cells. Results of this study provide a theoretical basis for the medical application of ellagic acid from pomegranate rind in cancer prevention and treatment.

## 2. Results

### 2.1. Identification of β-CD-Ellagic Acid Microspheres by SEM

**Figure 1:**
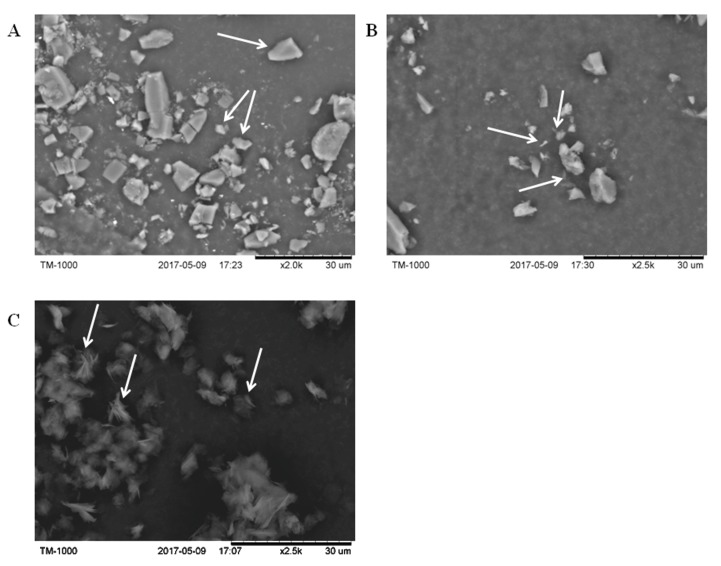
Scanning electron microscopy (SEM) images of β-cyclodextrin (β-CD)-ellagic acid microspheres. (**A**) β-CD; (**B**) ellagic acid; (**C**) β-CD-ellagic acid microspheres.

β-CD (Figure 1A) exhibited an irregular granular structure with a length of 5 ± 3.32 μm, as indicated by the arrows (*n* = 20). The pomegranate peel ellagic acid (Figure 1B) was a needle-shaped or short rod-like crystal structure with a length of 3.20 ± 0.78 μm, as indicated by the arrows (*n* = 20). The β-CD-ellagic acid complex (Figure 1C) was spherical with a size of 6.12 ± 3.27 μm, as indicated by the arrows (*n* = 30). Needle-like ellagic acids were dispersed on the surface or interior of the spherical particles, and it was speculated that β-CD-ellagic acid microspheres formed.

### 2.2. Identification of β-CD-Ellagic Acid Microspheres by FTIR

**Figure 2:**
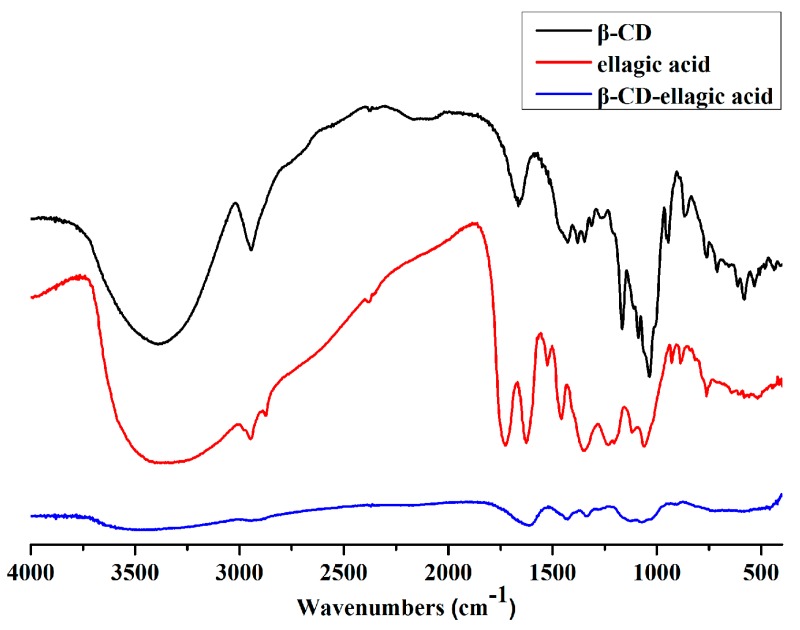
Infrared (IR) spectrum of β-CD-ellagic acid microspheres.

In β-CD, the infrared (IR) characteristic peak of O–H was at 3390 cm^−1^, the IR characteristic peak of C–H was at 2944 cm^−1^, and the IR characteristic peak of C–O was at 1035 cm^−1^. In the IR curve of ellagic acid, 1525 cm^−1^ and 1458 cm^−1^ were the stretching vibration peaks of the benzene ring skeleton; 3374 cm^−1^ was the O–H stretching vibration absorption peak; and 17.26 cm^−1^ and 1625 cm^−1^ were the absorption peaks of the carboxyl group. The IR spectra of β-CD-ellagic acid complexes was quite different from β-CD and ellagic acid, and the intensity of the characteristic stretching vibration peaks obviously decreased. However, the position of characteristic absorption peaks of β-CD-ellagic acid complex were similar with those of β-CD at 2944 cm^−1^ (C–H) and 1037 cm^−1^ (C–O). Meanwhile, the position of characteristic absorption peaks of the complex were similar to those of ellagic acid at 1627 cm^−1^ (benzene ring) and 1656 cm^−1^ (C=O). This finding showed that β-CD-ellagic acid microspheres had indeed formed, and the structure of the complexes was very dense (Figure 2).

### 2.3. Determination of Ellagic Acid Release Rates from Ellagic Acid Microspheres

**Figure 3:**
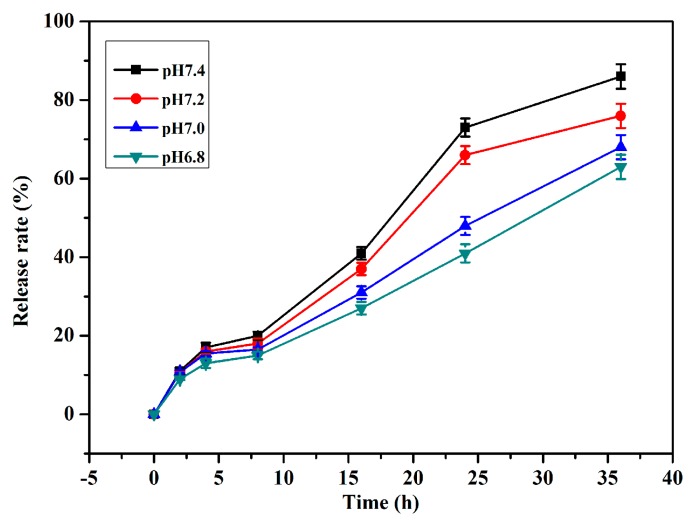
Release of ellagic acid from β-CD-ellagic acid microspheres at pH 7.4, 7.2, 7.0, 6.8.

The encapsulation efficiency of ellagic acid in β-CD-ellagic acid microspheres was 35.1%, and the molar ratio of β-CD and ellagic acid was 1:2. At pH 7.4 the slow release stage of ellagic acid from the microspheres was at 4–8 h, and only 3% of ellagic acid was released. The rapid release stage was at 8–24 h, and the release rate reached 73%. The slow release stage occurred again at 24–36 h, and another 13% of ellagic acid was released. The slow release of ellagic acid from the microspheres also indicated the successful preparation of β-CD-ellagic acid microspheres. The decrease of pH could inhibit the release of ellagic acid from the complex, and the most significant effect was at the rapid release phase of 8–24 h (Figure 3).

### 2.4. Effect of Ellagic Acid Microspheres on Tumor Cell Proliferation

Compared with the control group, 0.1 g/L of ellagic microspheres inhibited the proliferation of HepG2 cells after 6 h of treatment, and the cell proliferation significantly decreased after 12 h. A total of 0.3 g/L ellagic microspheres remarkably inhibited tumor cell proliferation at 12 h, and 0.5 g/L of ellagic acid microspheres remarkably inhibited tumor cell proliferation at 6 h. The inhibition of microspheres on tumor cell proliferation was in a dose- and time-dependent manner (Table 1).

**Figure 4:**
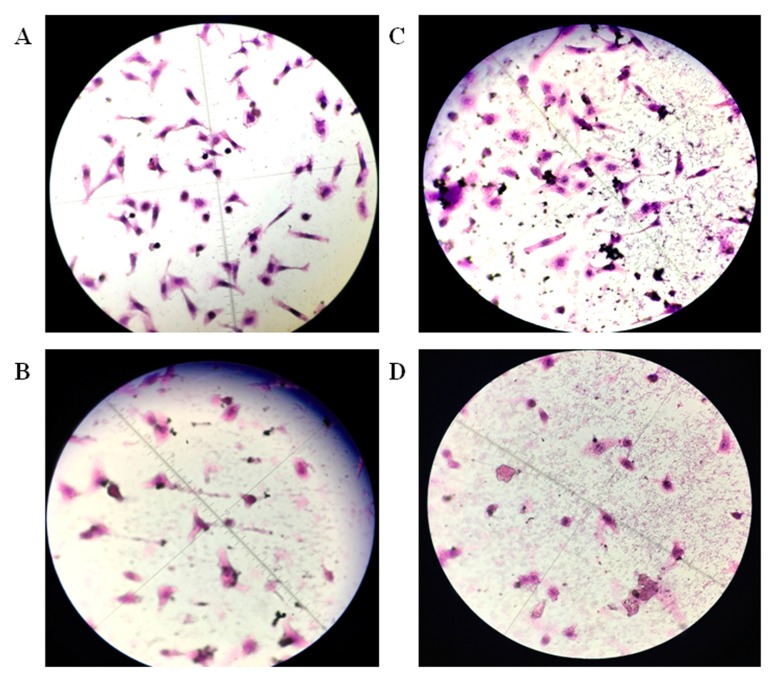
Hematoxylin-eosin (HE) staining to observe the effect of different concentrations of ellagic acid microspheres on HepG2 cell proliferation: (**A**) Control group; (**B**) 0.1 g/L ellagic acid microspheres; (**C**) 0.3 g/L ellagic acid microspheres; (**D**) 0.5 g/L ellagic acid microspheres (100×).

As shown in Figure 4A, tumor cells in the control group had good shape and were numerous. The nuclei were stained purple with a distinct boundary with the cytoplasm. Meanwhile, the cytoplasm was full and red. After adding ellagic acid microspheres, the cytoplasm shrank evidently, and the nuclei dispersed. Cell apoptosis occurred, and the number of cells decreased. With the increasing concentration of ellagic acid microspheres, cell contraction increased, and the number of cells decreased significantly. Ellagic acid microspheres exhibited an inhibitory effect on tumor cell proliferation, and a concentration-response relationship existed (Figure 4B–D).

### 2.5. DNA Damage of Tumor Cells Caused by Ellagic Acid Microspheres

**Figure 5:**
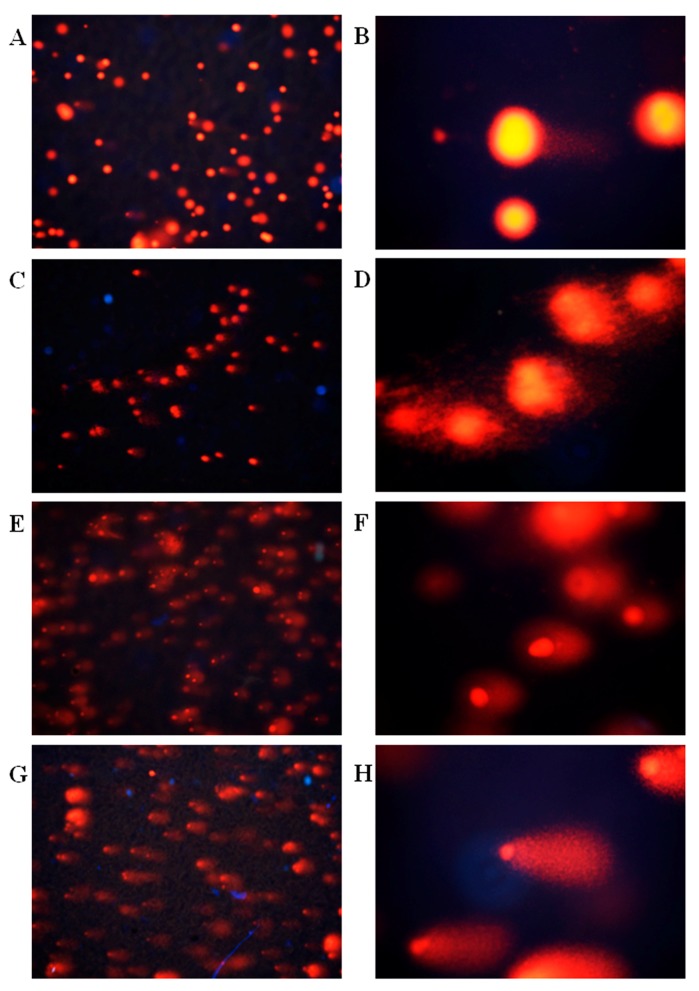
Effects of different concentrations of ellagic acid microspheres on HepG2 cells: (**A**,**B**) control group; (**C**,**D**) 0.1 g/L ellagic acid microspheres; (**E**,**F**) 0.3 g/L ellagic acid microspheres; (**G**,**H**) 0.5 g/L ellagic acid microspheres (**A**,**C**,**E**,**G**, 100×; **B**,**D**,**F**,**H** 400×).

Under the fluorescence microscope, DNA was stained orange by propidium iodide (PI), and cells with normal DNA had round fluorescence head (Figure 5A,B), but cells that had damaged DNA were cometary. The more fragments the DNA had, the longer the comet’s tail. In the control group, the DNA of tumor cells was complete, and the tumor cell appeared like a round bright spot. In the ellagic acid microsphere groups, different levels of DNA damage occurred, thereby resulting in a comet trailing tail phenomenon. The length of the comet’s tail corresponded to the concentration of ellagic acid microspheres. This result indicated that ellagic acid microspheres damaged the DNA of tumor cells in a dose-dependent manner.

**Figure 6:**
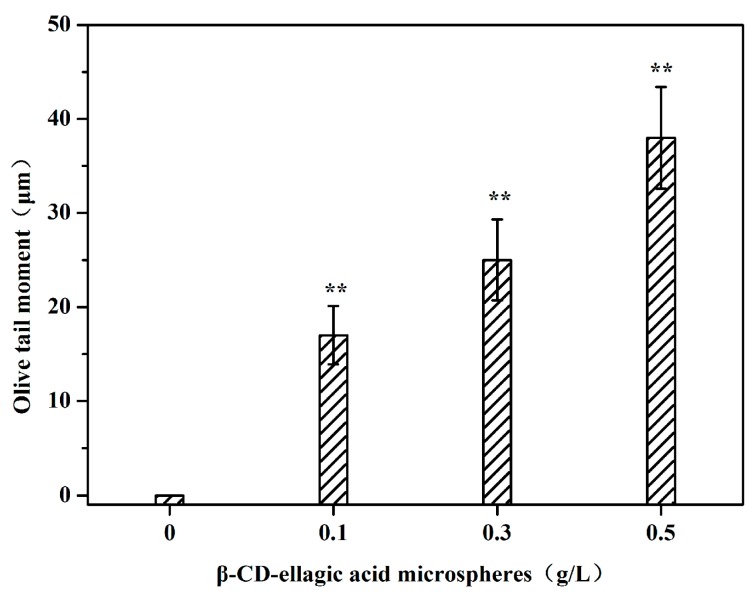
Effects of different concentrations of ellagic acid microspheres on the Olive tail moment of HepG2 cells. Significant differences between the ellagic acid microsphere groups and control group were tested using the Student-Newman-Keuls (S–N–K) test, ** *p* < 0.01.

The effects of ellagic acid microspheres on the Olive tail moment of DNA of in vitro cultured HepG2 cells are shown in Figure 6. When the concentration of ellagic acid microspheres increased, the Olive tail moment of the tumor cells exhibited a rising trend. When the dose of ellagic acid microspheres was 0.5 g/L, the DNA tail moment reached 38 μm. This result further illustrated the extent of tumor cell DNA damage caused by ellagic acid microspheres.

### 2.6. The mRNA expression of p53 Caused by Ellagic Acid Microspheres

**Figure 7:**
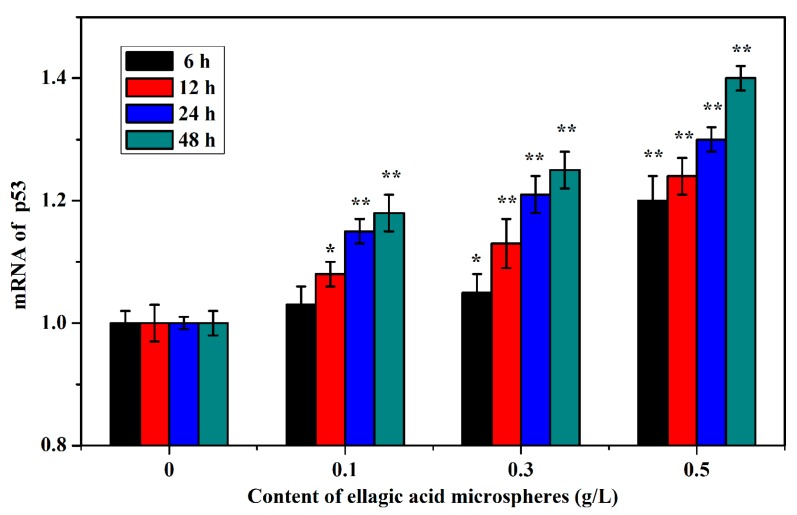
Effects of different concentrations of ellagic acid microspheres on the expression of p53 in HepG2 cells. Significant differences between the ellagic acid microsphere groups and control group were tested using the S–N–K test, * *p* < 0.05 and ** *p* < 0.01.

Compared with the control group, 0.1 g/L of ellagic microspheres upregulated the expression of p53 gene in HepG2 cells after 12 h of treatment, and the expression of p53 significantly increased after 24 h. A total of 0.3 g/L ellagic microspheres remarkably upregulated the expression of p53 at 12 h, and 0.5 g/L of ellagic acid microspheres remarkably upregulated the expression of p53 at 6 h. The upregulated expression of p53 was in a dose- and time-dependent manner (Figure 7).

## 3. Discussion

The ellagic acid from pomegranate peel is one of the most important natural active components of pomegranate. In this study, ellagic acid was enclosed effectively by β-CD to form ellagic acid microspheres. Identified using SEM and IR, the ellagic acid microspheres were successfully prepared, and ellagic acid in the microspheres could be released slowly. Inclusion compound microspheres enhanced the stability of the ellagic acid and also conferred the ellagic acid with more functions and excellent properties, such as heat resistance, light resistance, easy storage, and convenient transportation [20,21,22]. The size of the β-CD-ellagic acid complex averaged 6.12 μm by SEM; it did not reach a more microscopic size. The possibility was that the size of pomegranate peel ellagic acid reached micron size, and the prepared inclusion compound could only be larger. The variation in size of the β-CD-ellagic acid complex was large, and it was related to the structure of the complex. After the formation of the β-CD-ellagic acid inclusion, the inclusions could form polymers, and the different number of inclusions in the polymer determined the large variation in size of the β-CD-ellagic acid complex. The release of ellagic acid from the β-CD-ellagic acid complex depended on the structure of ellagic acid complex and pH. 0–4 h and 8–24 h were the fast release phases, and ellagic acid was released from free inclusions. The slow release phase lasted between 4–8 h, and free ellagic acid inclusions were released from polymers. Ellagic acid is slightly soluble in water, but has a good solubility in alkaline solution. Thus, the dissolution of ellagic acid was reduced when the pH decreased. Meanwhile, the release of ellagic acid from the complex was inhibited.

In cell experiments, ellagic acid microspheres inhibited the proliferation of human hepatoma cells. Greater the concentrations of ellagic acid microspheres and longer the treatment time reduced the cell proliferation rate, thereby showing a dose- and time-dependent correlation. In the low concentrations and at the slow release phase, the ellagic acid microspheres had a little effect on cell proliferation. However, in the fast release stage, the ellagic acid microspheres had considerable effect on cell proliferation. Hence, the effect of ellagic acid microspheres on tumor cells was consistent with the release of ellagic acid. Under high concentrations of ellagic acid microspheres, even in the slow release stage, the total release of ellagic acid was larger; thus, the inhibition on tumor cells was significant. Results of the single-cell gel electrophoresis and tail moment showed that the inhibition of tumor cell proliferation by ellagic acid microspheres was due to the damage of tumor cell DNA. A study reported that ellagic acid significantly reduced the proliferation and induced the apoptosis of tumor cells as evidenced by chromosomal DNA degradation and cell apoptosis appearance. Besides the progressive, time- and dose-dependent increase in DNA degradation, an increase in hypodiploid DNA content and significant time-dependent nuclear fragmentation were also confirmed [23]. Our results were consistent with their conclusion, and we found the upregulated expression of p53 gene might play an important role in the inhibition of ellagic acid microspheres on HepG2 cell proliferation. Its mechanism might be also related to the decreasing nuclear factor kappa B (NF-B) activity, thereby activating the mitochondrial death pathway, which is associated with the loss of mitochondrial membrane potential, cytochrome C release, and caspase-3 activation [24].

Ellagic acid is an effective component in pomegranate peel, and its extraction rate is 13.69% [25]. To overcome the bioavailability issues, many researchers developed slow release systems, such as the chitosan-glycerol phosphate in situ gelling system for the sustained subcutaneous delivery of ellagic acid [26], ellagic acid-loaded poly(d,l-lactide-coglycolide) nanoparticles for oral administration [27], and Eudragit P-4135F (a new pH-sensitive polymer) to deliver ellagic acid to the lower small intestine in rats [28]. A large number of microspheres, liposomes, nanoparticles, and polymeric implantable devices are emerging as alternatives for delivering therapeutic concentrations of ellagic acid into the systemic circulation; therefore, the bioavailability of ellagic acid has improved [29]. β-CD-ellagic acid microspheres can control the release of ellagic acid from the complex to ensure better compatibility, greater possibility of entering the cell, slower infiltration rate, and greater effect on the cell. The preparation of ellagic acid microspheres may also have a targeting effect, resulting in an accurate tumor targeting [30]. With the development of research on pomegranate peel ellagic acid microspheres and its application in tumor treatment, the comprehensive exploitation and utilization of pomegranate will be further developed.

## 4. Material and Methods

### 4.1. Cell Line and Reagents 

Human liver cancer cell line HepG2 (ATCC HB-8065). β-CD (CAS#7585399, purity > 98%, Sangon Biotech, Shanghai, China). Ellagic acid (isolated and purified from pomegranate rind in our own laboratory, purity >98%). Standard products of ellagic acid (purity > 98%, YuanYe Biotechnology Co., Ltd., Shanghai, China). DMEM F12 high-sugar culture medium (HyClone, Logan City, UT, USA). Fetal bovine serum, penicillin, streptomycin, and trypsin (Gibco, Grand Island, NY, USA); HE staining kit (Solarbio, Beijing, China). All other reagents are analytical reagents.

### 4.2. Preparation of β-CD–Ellagic Acid Microspheres

A total of 10 g of β-CD was dissolved in 400 mL distilled water for a saturated aqueous solution. Meanwhile, 2.5 g of pomegranate rind ellagic acid was dissolved in 20 mL of ethanol, and the ellagic acid solution was slowly trickled into the β-CD saturated solution. The mixture was stirred on a magnetic blender at room temperature for 2 h, chilled to room temperature, placed in a refrigerator overnight, and then freeze-dried using a desktop vacuum freeze-drying machine (Jingfu Instrument Co., Ltd., Shanghai, China) for several hours. The spongy compound was obtained by grinding and pulverizing.

### 4.3. SEM Analysis

First, black conductive adhesive was placed on the sample table. Then, β-CD, ellagic acid, and β-CD-ellagic acid microspheres were sprinkled on the conductive adhesive, respectively. The morphology of the above substances was observed using the desktop scanning electron microscope TM-1000 (Hitachi, Tokyo, Japan). The acceleration voltage was 15 kV, the scanning range was 6.5 mm, and the scanning speed was 3 (slow).

### 4.4. FTIR Spectroscopy

FTIR spectra were recorded for β-CD, ellagic acid, and β-CD-ellagic acid microspheres by KBr disc using the IR spectrometer FTIR-7600 (Lambda, Spring Hill, Australia). A 1 mg sample was mixed with 100 mg KBr; then, the mixture was laminated with DF-4 sheeting out roller (Gangdong Technology Development Co., Ltd., Tianjin, China). The spectra were obtained with 80 scans per sample, ranging from 400 cm^−1^ to 4000 cm^−1^ at a resolution of 1 cm^−1^ by monitoring the IR absorbance.

### 4.5. Determination of Entrapment Efficiency and Release Rate of Ellagic Acid in Ellagic Acid Microspheres

Ellagic acid microspheres (200 mg) were ground in a mortar and subsequently suspended with 5 mL of ethanol. Ultrasonic treatment was sustained for 2 h, and precipitation was discarded. The supernatant was diluted with 50% ethanol solution, and the encapsulation efficiency was measured using HPLC (Waters 510, Milford, MA, USA) at 254 nm. A total of 20 mg of ellagic acid microspheres was suspended in 200 mL of phosphate buffer (PBS, 0.2 mol/L, pH 7.4, 7.2, 7.0, 6.8), bathing at 37 °C for 6, 12, 24, and 36 h, and the release rates of ellagic acid from ellagic acid microspheres were also measured using HPLC [31,32].

### 4.6. Effect of Ellagic Acid Microspheres on HepG2 Cell Proliferation

The concentration of logarithmic growth phase cells was regulated to 1 × 10^5^/mL, and tumor cells were inoculated in 96-well plates. HepG2 cells were cultured in a CO_2_ incubator (371, Thermo, Waltham, MA, USA) for 24 h; the cells were treated with 0.1, 0.3, and 0.5 g/L ellagic acid microspheres for 6, 12, 24, and 48 h. A total of 10 μL of 0.5 mg/mL MTT (Sigma, Santa Clara, CA, USA) solution was added in the sample holes, and another 4 h culture was carried out. After discarding the medium in the sample holes, 100 μL of DMSO was added; gentle shaking for 5 min took place. The absorbance at 570 nm of each hole was detected using Multiskan GO (Thermo, Waltham, MA, USA). Cells were washed thrice with phosphate buffered solution (PBS) and fixed for 30 min with 4% formaldehyde. Cells were stained with hematoxylin for 20 min and with eosin for 2 min after 30 s differentiation. Finally, the cells were observed under an invert microscope (Olympus, Tokyo, Japan) [33,34].

### 4.7. Single-Cell Electrophoresis 

Agarose 1% (100 μL) with normal melting point (NMA) was spread on frosted glass slide and flattened with cover glass. Agarose coagulated at low temperature, and air bubble formation was avoided. A total of 10 μL of tumor cells (1 × 10^5^/mL) was mixed with 1% agarose at 90 μL with low melting point. The mixture was spread on NMA; the cover glass was removed after coagulation. Cells were placed in a precooled lysate for lysis at 4 °C for 2 h. Unwinding took place three times thereafter for 5 min each in a precooled deionized water. The electrophoresis voltage was 25 V, the current was 300 mA, and the time was 30 min. The samples were placed in a neutralization solution for 15 min. After staining with 0.5 mg/L propyl iodide solution, the DNA was observed using a fluorescence microscope (Olympus BX53, Tokyo Metropolis, Japan), and the images were analyzed using the Comet Assay Software Project software (CASP 1.2.3, Wroclaw, Poland) [34,35].

### 4.8. Reverse Transcription-Quantitative Polymerase Chain Reaction (qPCR) Analysis

The RNA extracted from HepG2 cells treated with β-CD-ellagic acid microspheres (0.1, 0.3, and 0.5 g/L) for 6, 12, 24, 48 h, as described above, and the RNA extracted from cells without any treatment was used as the control. The purity identification and quantification of RNA were performed with GENE QUANT 1300 (GE Healthcare, Little Chalfont, UK). RNA was transcribed to cDNA using a RevertAid First strain cDNA Synthesis kit (#K1622, Fermantas, EU, Waltham, MA, USA). qPCR was performed with SYBR Green I (Applied Biosystems, FosterCity, CA, USA). An absolute quantification method was used to quantify the p53 genes, and the β-actin was used as an internal control. The primers for p53 were CAGCCAAGTCTGTGACTTGCACGTAC and CTATGTCGAAAAGTGTTTCTGTCATC, and the primers for β-actin were GCTCGTCGTCGACAACGGCTC and CAAACATGATCTGGGTCATCTTCTC.

### 4.9. Statistical Analysis

All experiments had three technical replicates. Data of independent experiments were expressed as the mean ± SD and analyzed with the SPSS 18.0. Significant differences were assessed using the Student’s *t* test. *p* < 0.05 was considered to be statistically significant.

## 5. Conclusions

In this study, we parceled pomegranate peel ellagic acid with β-CD, and the effects of the inclusion complex on the proliferation of HepG2 cells were observed. β-CD-ellagic acid microspheres were successfully prepared, and the inhibition of microspheres on tumor cell proliferation was controlled by a sustained slow. Results suggested that β-CD-ellagic acid microspheres have potential for clinical use in oncotherapy.

## Figures and Tables

**Table 1:** Effect of ellagic acid microspheres on HepG2 cell proliferation.

Dose (g/L)	Absorbance (A)				
	0 h	6 h	12 h	24 h	48 h
0	0.203 ± 0.016	0.232 ± 0.031	0.294 ± 0.026	0.321 ± 0.025	0.337 ± 0.033
0.1	0.212 ± 0.044	0.205 ± 0.021 *	0.186 ± 0.030 **	0.173 ± 0.019 **	0.161 ± 0.035 **
0.3	0.198 ± 0.031	0.174 ± 0.042 *	0.122 ± 0.020 **	0.093 ± 0.015 **	0.086 ± 0.014 **
0.5	0.204 ± 0.025	0.095 ± 0.018 **	0.079 ± 0.016 **	0.067 ± 0.010 **	0.051 ± 0.009 **

Compared with control group, * *p* < 0.05, ** *p* < 0.01.
